# Screening of white-rot fungi manganese peroxidases: a comparison between the specific activities of the enzyme from different native producers

**DOI:** 10.1186/2191-0855-2-62

**Published:** 2012-11-29

**Authors:** Juho Järvinen, Sanna Taskila, Ritva Isomäki, Heikki Ojamo

**Affiliations:** 1 Bioprocess Engineering Laboratory, Department of Process and Environmental Engineering, University of Oulu, P. O. Box 4300, Oulu, FI-90014, Finland

**Keywords:** White-rot fungi, Manganese peroxidase, Lignin degradation, Lignocellulose, Enzyme purification

## Abstract

In this study manganese peroxidase (MnP) enzymes from selected white-rot fungi were isolated and compared for potential future recombinant production. White-rot fungi were cultivated in small-scale in liquid media and a simplified process was established for the purification of extracellular enzymes.

Five lignin degrading organisms were selected (*Bjerkandera sp., Phanerochaete* (*P.*) *chrysosporium*, *Physisporinus* (*P.*) *rivulosus*, *Phlebia* (*P.*) *radiata* and *Phlebia sp. Nf* b19) and studied for MnP production in small-scale. Extracellular MnP activity was followed and cultivations were harvested at proximity of the peak activity. The production of MnPs varied in different organisms but was clearly regulated by inducing liquid media components (Mn2+, veratryl alcohol and malonate). In total 8 different MnP isoforms were purified.

Results of this study reinforce the conception that MnPs from distinct organisms differ substantially in their properties. Production of the extracellular enzyme in general did not reach a substantial level. This further suggests that these native producers are not suitable for industrial scale production of the enzyme. The highest specific activities were observed with MnPs from *P. chrysosporium* (200 U mg-1), *Phlebia sp. Nf* b19 (55 U mg-1) and *P. rivulosus* (89 U mg-1) and these MnPs are considered as the most potential candidates for further studies. The molecular weight of the purified MnPs was estimated to be between 45–50 kDa.

## Introduction

At present lignocellulose is a major raw material for forestry, pulp and paper industry and the emerging second generation biofuel production. Among cellulose and hemicellulose, lignin is a major component of lignocellulosic biomass and largely responsible for its strength. Inside the Northern coniferous forest belt the importance of lignin utilization is stressed in wood-based biorefineries due to high amounts of lignin in softwoods (Li *et al.*^ 2009^).

Lignin is a heterogeneous, branched and complex polymer consisting of phenylalanine-derived aromatic subunits (Whetten &Sederoff 1995). Because of its recalcitrance, lignin complicates the utilization of biomass polysaccharides in biorefineries and increases the energy consumption in mechanical pulping (Jiang *et al.*^2008^a). In nature one group of organisms, the *basidiomycetous* fungi, are able to effectively degrade lignin by employing a family of lignin degrading enzymes. These organisms can be divided into wood-colonizing white-rot fungi and soil litter-decomposing fungi. Fungal attack on lignin is attributed to certain secreted nonspecific oxidoreductases, which produce low molecular weight mediators able to intrude recalcitrant biopolymers. The family of extracellular ligninolytic enzymes typically includes lignin peroxidases (LiP, EC 1.11.1.14), laccases (EC 1.10.3.2), manganese peroxidases (MnP, EC 1.11.1.13), versatile peroxidases (VP, EC 1.11.1.16) and other accessory enzymes.

Out of these enzymes MnP is thought to play the most crucial role in lignin degradation, as it is found in all lignin degrading fungi white-rot fungi. This heme protein belongs to the commonly occurring class II peroxidase group in *basidiomycetous* fungi and has a highly specific Mn2+ −binding site. In the binding site of classical long MnPs there are three amino-acid residues (two Glu and one Asp) while several fungal Mn2+ −oxidizing enzymes with an additional tryptophan residue on the enzyme surface have been found. These enzymes are called VPs or hybrid MnPs, bearing resemblance to LiPs and able to perform oxidation though long-range electron transfer as well. The evolution of these class II peroxidases appears to be closely related to each other and even consistent with the sharp decline in coal accumulation rate during the Permo-Carboniferous period. Lignin is the main precursor for coal (Hofrichter *et al. *2010;Floudas *et al.*^2012^). MnP catalyses the oxidation of Mn2+ −ions to highly reactive Mn3+−ions. Chelated Mn3+ in turn act as low molecular weight mediators that are able to attack phenolic structures. MnP is able to cause substantial depolymerization if lignin in *in vitro* biomass treatments (Hofrichter 2002;Hofrichter *et al.*^2001^;Maijala *et al.*^2008^).

Potential applications for MnP include biomechanical pulping, pulp bleaching, dye decolorization, bioremediation and production of high-value chemicals from residual lignin from biorefineries and pulp and paper side-streams (Maijala *et al.*^2008^;Moreira *et al.*^2003^;Moreira *et al.*^2001^;Xu *et al.*^2010^a and 2010b;Paice *et al.*^1993^;Susla *et al.*^2008^;Hofrichter *et al.*^1998^;Sack *et al.*^1997^). Applications of MnP are limited due to slow growth and low productivity of native enzyme producers and lack of an efficient recombinant production process (Hofrichter 2002;Jiang *et al.*^2008^a).

The production of lignolytic enzymes and its regulation has been intensively studied in various lignin degrading fungi (Bonnarme &Jeffries 1990;Hakala *et al.*^2006^; Jiménez-Tobon *et al.*^2003^;Kamitsuji *et al.*^2004^;Lankinen *et al.*^2005^; Martínez *et al.*^1996^;Moilanen *et al.*^1996^;Nuske *et al.*^2002^; Perie &Gold 1991;Palma *et al.*^2000^;Petruccioli *et al.*^2009^;Schneegab *et al.*^1997^; Swamy &Ramsay 1999;Steffen *et al.*^2002^;Susla *et al.*^2008^;Silva *et al.*^2008^;Sklenar *et al.*^2010^;Singh *et al.*^2011^; Taboada-Puig *et al.*^2011^;Vares *et al.*^1995^;Wang *et al.*^2001^;Wang *et al.*^2008^) and novel bacterial strains (Bharagava *et al.*^2009^; Mishra &Thakur 2010;Yadav *et al.*^2011^). Recombinant production has been studied in filamentous fungi (Conesa *et al.*^2000^;Irie *et al.*^2001^;Li *et al.*^2001^;Mayfield *et al.*^1994^;Stewart *et al.*^1996^), yeasts (Jiang *et al.*^2008^a), bacterial (Whitwam &Tien 1996) and insect (Johnson *et al.*^1992^*;*Pease *et al.*^1991^) hosts with successful production but modest yields of active enzyme. MnP from *P. chrysosporium* has been the target of most recombinant studies, which however suffer from unsuccessful posttranslational protein modification and the need for exogenous heme in high concentrations (Jiang *et al.*2008a).

Furthermore, industrially robust enzymes need to have high stability in demanding process conditions which promotes the need for screening novel enzymes and enzyme modification. Multiple crystal structures and molecular models based on gene sequences have been published for MnP and VP (*e.g.*Sundaramoorthy *et al.*^1997^;Sutherland *et al.*^1997^andMoreira *et al.*^2005^). The effects of disulfide bonds and calcium-ions on the stability of MnP have been studied byReading *et al.* (2001) andSutherland *et al.* (1997), respectively. As a result of enzyme modification, a mutant MnP with one additional disulfide bond had increased stability in alkaline (pH 8) conditions (Reading *et al.*^2001^). Another strategy is to screen for native MnPs with improved properties regarding thermostability, specific activity or pH optimum and stability (Petruccioli *et al.*^2009^and Urek &Pazarlioglu 2004). Increased specific activity (U mg-1 protein) of the selected protein would also increase the profitability of the recombinant process. Independent of the strategy the bottleneck is the establishment of an efficient recombinant production.

The aim of the present study was to screen MnPs from promising candidates of lignin degrading fungi for future recombinant production. Specific activities of MnPs were determined after cultivation and purification procedures. Isoforms of MnP from different organisms differ substantially and screening for novel MnP enzymes is beneficial for designing applications and developing a production process capable of producing the enzyme at an economically feasible cost. However high-throughput screening is challenging due to low enzyme yields and diverse regulation of MnP isoenzymes in different native hosts.

## Materials and methods

### Fungi and culture conditions

White-rot fungi strains *Bjerkandera sp.* BEL LLP4 (D-00810) and *P. radiata* Hatakka & Pirhonen strain 79 (ATCC 64658, D-84236) were ordered from the VTT Technical Research Centre of Finland culture collection, Finland. Strains *P. chrysosporium* (ATCC 24725, DSM 6909), *Phlebia sp. Nf* b19 (ATCC 201144, DSM 11239) and *P. rivulosus* T241i (DSM 14618) were ordered from the Leibniz institute DSMZ-German collection of microorganisms and cell cultures GmbH, Germany. *Phlebia sp. Nf* b19 was originally identified as *Nematoloma frowardii* species but reassigned to the family Corticiaceae and genus *Phlebia* based on a molecular level study by Hildén *et al.* (2008).

The fungi were maintained on solid agar plates. The agars used were 0.4% potato 2% dextrose agar (Difco, USA) for *P. radiata*, 3% malt extract-peptone agar (Merck, Germany) for *P. chrysosporium, Phlebia sp. Nf* b19 and *P. rivulosus* and 3% malt agar (Difco, USA) for *Bjerkandera sp*. The plates were incubated for 2–10 days until covered with mycelium. Plates were stored at 4°C and replated monthly.

The inocula were prepared by suspending the mycelia from one plate to sterile 0.9% (w/v) Sodium chloride. One plate was used to inoculate two parallel fungi cultivations.

The production of extracellular MnP in each organism was followed in two parallel cultivations in 1 L shake flasks containing 300 mL of liquid media with 90 rpm agitation. Cultivation temperatures were 37°C for *P. chrysosporium*, 25°C for *Phlebia sp. Nf* b19 and 28°C for all other white-rot fungi strains. *P. radiata* was grown in low-nitrogen ADMS medium pH 4.5 (Hatakka & Uusi-Rauva 1983) with 1% glucose, 0.05% (w/v) Tween 80 and 1 mM veratryl alcohol (VA, 3,4-dimethoxybenzyl alcohol). Supplements 180 μM Mn2+ and 10mM Sodium malonate were added on the 4th day. *P. rivulosus* was grown in low-nitrogen ADMS medium pH 4.5 (Hatakka & Uusi-Rauva 1983) with 1% glucose and 0.05% (w/v) Tween 20. Supplements 24 μM Mn2+ and 0.36 mM VA were added on the 4th day. *P. chrysosporium* was grown in Kirk’s medium pH 4.5 (Urek &Pazarlioglu 2004; Tien &Kirk 1988and Bonnarme &Jeffries 1990) with 1% glucose and 0.05% (w/v) Tween 80. Supplements 728 μM Mn2+ and 0.36 mM VA were added on the 4th day. *Bjerkandera sp.* was grown in Kirk’s medium pH 4.5 (Palma *et al.*^2000^) with 1% glucose and 0.05% (w/v) Tween 80, 235 μM Mn2+ and 0.36 mM VA. *Phlebia sp. Nf* b19 was grown in a glucose-yeast extract medium pH 4.5 (Nuske *et al.*^2002^) with 0.5% glucose, 0.03% yeast extract and 200 μM Mn2+. Cultivations were continued until extracellular MnP activity was detected and it started to decline.

### Sampling

1mL samples were taken every 24 h from each flask. Samples were centrifuged to remove cellmass (16000 × g, 4°C). The supernatant was used for determination of MnP activity and glucose and total protein concentrations. All of the cultivations were performed in duplicate and the presented results are average values.

### Enzyme assay and analytical procedures

Glucose concentration during cultivation was determined using the YSI 2700 SELECT™ (YSI limited, UK, Hampshire) biosensor.

MnP activity was determined spectrophotometrically at 270 nm by following the formation of Mn3+ −malonate complex at pH 4.5 in 50 mM sodium malonate buffer with 0.5 mM MnSO4. The reaction was initiated by adding H2O2 to the final concentration of 0.1 mM (Wariishi *et al.*^1992^). The reaction was followed for 30 sec at room temperature. ΔAbs min-1 was converted to U L-1 using an extinction coefficient of 11590 M-1cm-1. Genesys 10UV spectrometer (Thermo Scientific, USA) was used in all the measurements with semi-micro 1.4 mL UV quartz cuvettes (Sigma-aldrich, Germany)

Total protein was determined with the Bio-Rad protein Assay (USA) using bovine serum albumin (BSA) as a standard. Measurements were made with Genesys 10UV spectrometer (Thermo Scientific, USA) at 595 nm wavelength.

Protein expression and purity was followed using sodium dodecyl sulfate polyacrylamide gel electrophoresis (SDS-PAGE) with silver nitrate staining. Ready-made Bio-Rad (USA) ready GelTM precast gels (12% Tris–HCl) were used with Bio-Rad (USA) prestained low range protein standards.

### Purification of manganese peroxidases

Cellmass was separated from the culture fluids by centrifugation at 16000 × g, 4°C (Avanti J series centrifuge, Beckman Coulter Inc, USA). Supernatant was then filtered through a Whatman grade 1 filter paper (11μm, Whatman, UK). Filtrate was then stored in −20°C until further processing.

Concentration of the culture fluids from the screening cultivations was done by using Vivaspin 20 centrifugal concentrators with 10 kDa cut-off membranes (GE Healthcare, USA and Sartorius Stedim Biotech, Germany). Crude culture liquids were concentrated approximately tenfold by volume. Megafuge 1.0R (Heraeus, England) was used for centrifugation at 3400 × *g*, 4°C. Diafiltration was performed in Vivaspin tubes by filtering 20 mM Bis-TRIS-propane buffer (pH 6.2) through the membrane (three times the volume of the concentrate).

ÄKTAavantTM liquid chromatography system (GE Healthcare, USA) equipped with a strong anion exchange column (HiScreenTM CaptoTM Q, GE Healthcare, USA) was used for protein separation. The outflow was monitored at two wavelengths: 280 nm for protein and 405 nm for hemeprotein detection. The column was first equilibrated with 20 mM Bis-TRIS-propane buffer (pH 6.2) and proteins were eluted with a linear gradient of 0–1 M NaCl in the same buffer. 2 mL fractions were collected during the elution phase. The fractions were assayed for MnP activity and total protein. Peaks were pooled separately and stored at −20°C.

## Results

### MnP production in selected white-rot fungi

**Figure 1:**
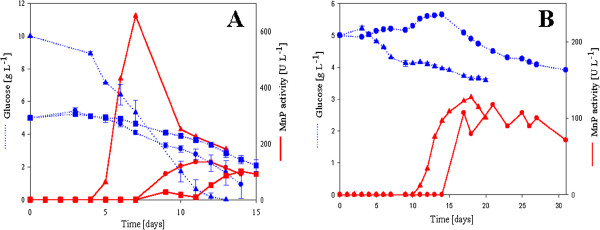
**Glucose consumption (dotted line) and MnP activity (solid line) by white-rot fungi during 300 mL batch cultivations.** Each strain was cultivated in specific conditions and media described in the materials section. Values are calculated averages of two parallel cultivations. Standard deviation of MnP activity in parallel cultures is on average ±40 UL^-1^. *Bjerkandera sp.* (**A**, blue and red triangles), *Phanerochaete chrysosporium* (**A**, blue and red circles), *Physisporinus rivulosus* (**A**, blue and red squares), *Phlebia radiata* (**B**, blue and red triangles), *Phlebia sp. Nf* b19 (**B**, blue and red circles). Harvesting point is designated by the end of sampling.

The activity (U l-1) of MnP enzyme produced and the time period it took to reach the maximal activity varied greatly between the selected white-rot fungi. A unifying trend of accelerated glucose consumption just before and during MnP production was observed in these batch cultivations (Figure 1A &1B). The MnP peak timing and maximal activity varied between 7–21 days and 100–800 U l-1, respectively. Fastest growing white-rot fungus in these experiments was *Bjerkandera sp.*, which also produced the highest activities. However, the MnP activity of *Bjerkandera sp.* increased and subsequently declined very sharply. These cultures were harvested 6 days after the MnP activity peak, which may have resulted in inactivated protein and lowered specific activity.

### Purification of MnP isoenzymes from crude culture filtrates

**Figure 2:**
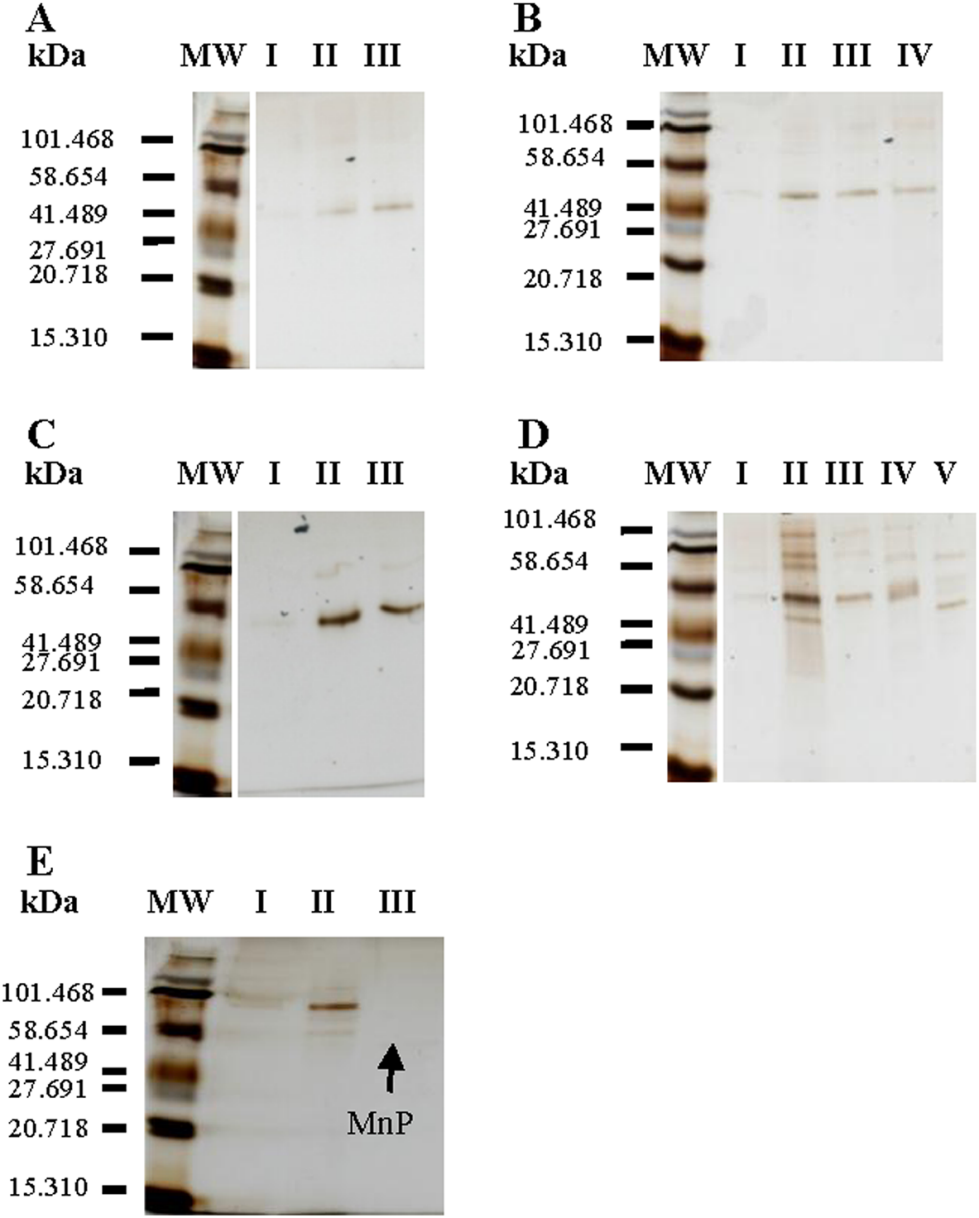
(A-E) Silver stained SDS-PAGE gels in corresponding order from the purification process with molecular weight protein standards (MW), crude extracellular fluids (I), concentrated samples (II) and MnP isoenzymes from MonoQ anion exchange column (III-V).

**Figure 3:**
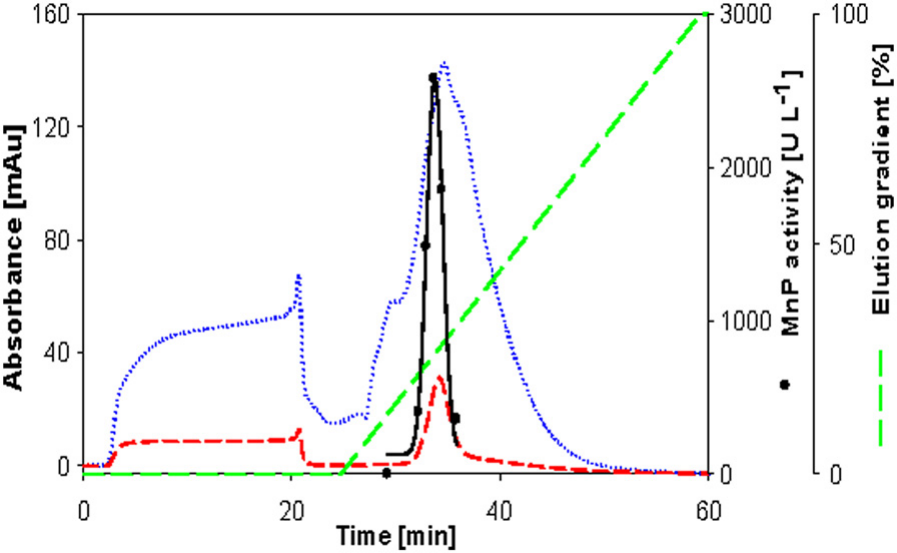
**Anion exchange chromatogram from the purification of MnP from *Bjerkandera sp.*** Dotted line is the absorbance at wavelength 208 nm; Short dash line is the absorbance at wavelength 405 nm; Long dash line is the concentration of the eluent in percentage.

**Figure 4:**
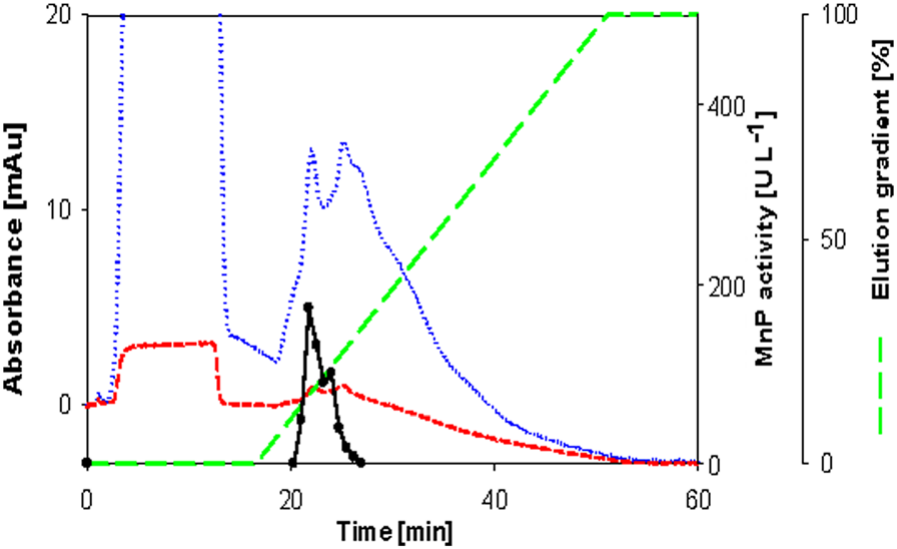
**Anion exchange chromatogram from the purification of MnP from *Phanerochaete chrysosporium.*** Symbols as in Figure 2.

**Figure 5:**
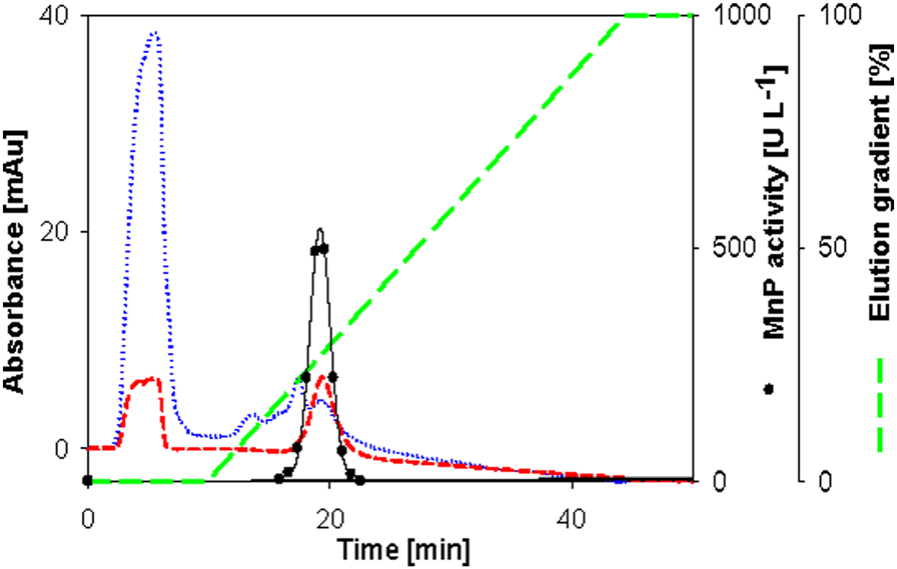
**Anion exchange chromatogram from the purification of MnP from *Physisporinus rivulosus.*** Symbols as in Figure 2.

**Figure 6:**
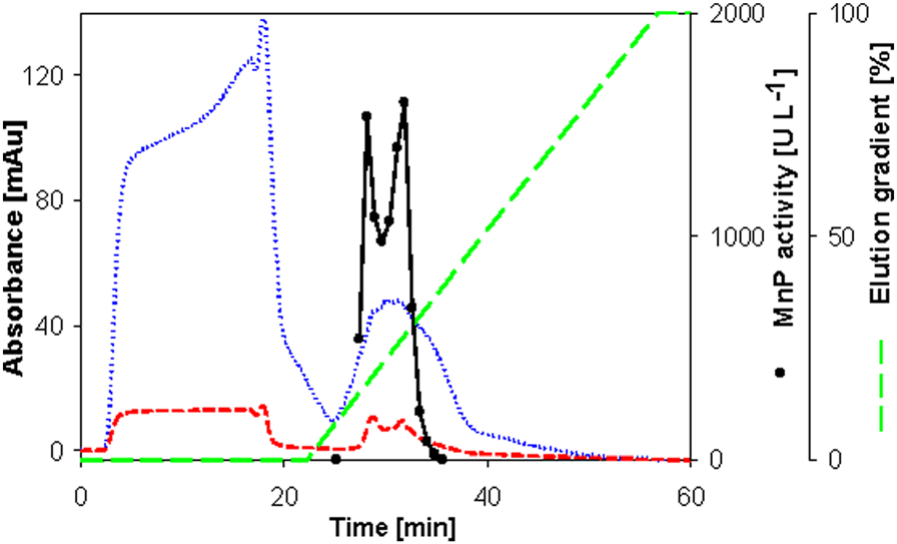
**Anion exchange chromatogram from the purification of MnP from *Phlebia radiata.*** Symbols as in Figure 2.

**Figure 7:**
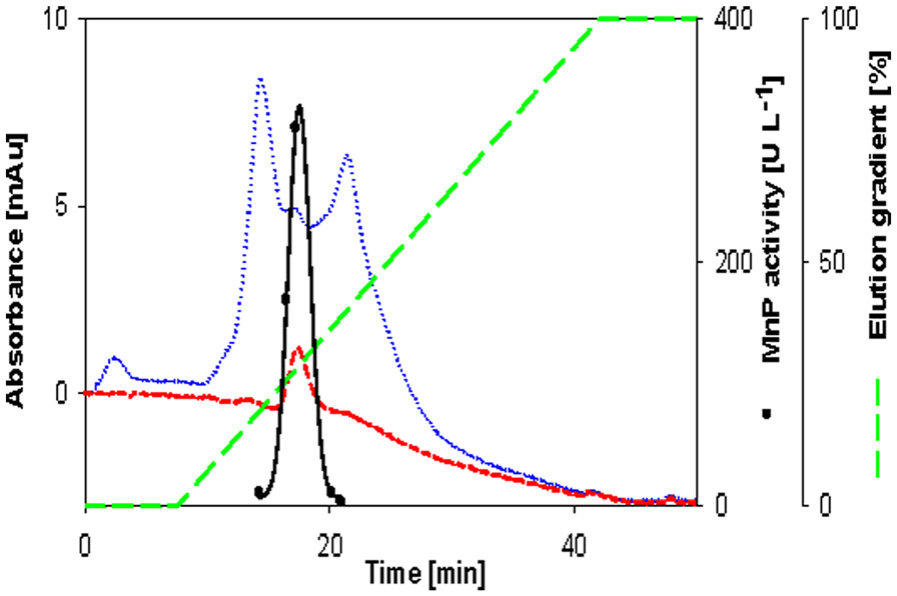
**Anion exchange chromatogram from the purification of MnP from *Phlebia sp. Nf *b19.** Symbols as in Figure 2.

Crude culture filtrates were concentrated and purified in a rapid purification scheme described in the materials section. Based on the SDS-PAGE gels (Figure 2A-E) MnP was the predominant protein produced in most of the cultures and highly purified in the pooled anion exchange fractions (Figures 3, 4, 5, 6, 7). In the *Phlebia sp. Nf* b19 culture supernatant and concentrate, a larger enzyme (around 70 kDa) was the predominant one. Based on the size, this is probably a laccase. Loss of MnP activity was high during the purification process. MnP activity was lost especially in the concentration step, but this is not relevant for the determination of specific activity. The role of enzyme inactivation in the purification procedure is minimized due to low kept temperatures (4°C) during each step. After the strong anion exchange (Mono Q column) one MnP isoenzyme for *Bjerkandera sp.* (Figure 3), *Phlebia sp. Nf* b19 (Figure 7) and *P. rivulosus* (Figure 5); two MnP isoenzymes for *P. chrysosporium* (Figure 4) and possibly three isoenzymes for *P. radiata* (Figure 6) were detected. All of the MnP active fractions showed absorbance peaks in the 280 nm wavelength for protein and in the 405 nm wavelength for the heme containing protein. Wavelenght 405 nm could easily be used for selecting fractions likely to show MnP activity. MnP proteins were eluted between 175 and 320 mM NaCl concentrations.

The molecular weight of the MnPs was between 45 and 50 kDa (Table 1). Specific activities of the MnPs were calculated on the basis of the activity measurements and measured total protein in the pooled active fractions. *P. chrysosporium* MnP1 had the highest specific activity (200 U mg-1). The second highest specific activity was observed for *P. rivulosus* (89 U mg-1). *Phlebia sp. Nf* b19 MnP had the specific activity of 55 U mg-1. Rest of the MnP isoenzymes had a specific activity below 40 U mg-1 (Table 1).

**Table 1:** **Purification of MnP isoenzymes from *Bjerkandera sp. *(A), *Phanerochaete chrysosporium *(B), *Physisporinus rivulosus *(C), *Phlebia radiata *(D) and *Phlebia sp. Nf *b19 (E)**

	**Activity [U]**	**Protein [mg]**	**Specific activity [U/mg]**	**Activity protein absorbance ratio [UL**^**-1**^**absorbance at 280nm**^**-1**^**]**	**Yield [%]**	**Purification fold**	**Size of MnP [kDa]**
A							
Culture fluid	38.0	2.9	13.3		100	1.0	
Ultrafiltration	19.8	2.6	7.6		52.2	0.6	
Mono Q BSMnP1	15.3	0.5	**32.3**	18	40.2	2.4	**45**
B							
Culture fluid	38.7	1.447	26.7		100	1.0	
Ultrafiltration	6.9	0.403	17.0		17.7	0.6	
Mono Q PCMnP1	1.1	0.005	**200.5**	13	2.8	7.5	**45**
Mono Q PCMnP2	0.4	0.013	**33.0**	8	1.1	1.2	**45**
C							
Culture fluid	36.6	2.685	13.6		100	1.0	
Ultrafiltration	7.7	0.278	27.6		20.9	2.0	
Mono Q PRMnP1	2.3	0.026	**88.6**	122	6.2	6.5	**50**
D							
Culture fluid	52.0	4.669	11.2		100	1.0	
Ultrafiltration	24.6	2.713	9.1		47.2	0.8	
Mono QPradMnP1	5.8	0.245	**23.7**	33	11.1	2.1	**50**
Mono Q PradMnP2	4.0	0.199	**19.9**	21	7.6	1.8	**50**
Mono Q PradMnP3	7.2	0.234	**30.8**	33	13.8	2.8	**50**
E							
Culture fluid	7.1	0.671	10.6		100	1.0	
Ultrafiltration	3.4	0.511	6.6		46.9	0.6	
Mono Q NFMnP1	0.7	0.012	**55.2**	67	9.3	5.2	**49**

Preliminary results of *in vitro* enzymatic processing of milled pinewood sawdust with crude MnP concentrates showed up to 17% (data not shown) decrease in Klason lignin content and benefitted from the use of co-oxidants (Tween 80 and glutathione) in some cases.

## Discussion

These results show that a simplified purification process is capable of producing comparable specific activity results for the screening of novel MnP enzymes. Culture supernatant concentration and anion exchange chromatography using a Mono Q column was enough to purify the enzyme to a high degree. Although the initial enzyme amounts were low and the losses during concentration were quite high, these results provide a basis for enzyme ranking. Based on these results there are significant differences in the specific activities of MnP enzymes from different white-rot fungi. Out of the white-rot fungi used in this study *P. chrysosporium*, *P. rivulosus* and *Phlebia sp. Nf* b19 MnPs were the most promising. For *P. rivulosus* and *Phlebia sp. Nf* b19 these results are also consistent with the MnP activity and protein absorbance (280 nm) ratio in anion exchange chromatography (Table 1). The MnP activity (U l-1) of *Bjerkandera sp.* had come down over 70% at the point of harvesting. If the lost activity is accounted as inactivated MnP in the total protein measurement, the specific activity could be at least three-fold higher. In addition to specific activity the stability of the purified MnP enzymes towards temperature, pH and inactivating compounds needs to be studied before candidates for recombinant production are selected. The reported specific activities are significantly lower than those previously reported for *P. chrysosporium* byUrek *et al.* (2004), *Bjerkandera sp.* byPalma *et al.* (2000) and Taboada-Puig *et al.* (2011) and for *Phlebia sp. Nf* b19 by Schneegaβ *et al.* (1997)

The production of MnP by native producers, as also observed in this study, is limited due to relatively low maximal enzyme activities, slow growth and the sensitivity of white-rot fungi towards shear forces, difficult induction strategies and low adaptivity to submerged fermentations. In this study MnP was in most cases induced with several supplements (Mn2+, VA, Tween, Sodium malonate). However, as reported byHakala *et al.* (2006), the regulation of different MnP isoforms can be largely dependent on the inducing compounds and nutrient (nitrogen and carbon) sources and amounts. This suggests that novel isoforms would be found by changing the culture conditions. Mn2+−ions are typically necessary inducers for MnP production although repressive effects of Mn2+−ions have also been reported (Martinez *et al.*^1996^). Although high activity level was not the focus in this study, the culture conditions and inducing strategies (see the materials section) for this study were selected on previous literature to maximize enzyme production. Specified effects of individual inducing components cannot be separated for any strain, but clearly no MnP was produced before inducing components were added.

In recombinant MnP processes with *Pichia pastoris* (Jiang *et al.*^2008^b), *Aspergillus oryzae* (Stewart *et al.*^1996^), *Aspergillus niger* (Conesa *et al.*^2000^) and *Echerichia coli* (Whitwam *et al.*^1995^; Whitwam &Tien 1996) production of high amounts of active protein has been a problem. These processes suffer from incorrect protein folding, insufficient heme synthesis and heme incorporation. However, MnP yield might be significantly increased with the right combination of recombinant enzyme, production host, promoter system, protein secretion system and optimized process conditions.Jiang *et al.* (2008b) also suspected that *P. pastoris* lacks the proper heme escorts and reseptors to transport exogenous heme to the site of MnP synthesis. This problem might be relieved by inserting the genes for such heme transport from *e.g. Shigella* (*S.*) *dysenteriae* (Mills &Payne 1995). *S. dysenteriae* is a known pathogen causing shigellosis, which would make the GMO rating of the recombinant strain difficult. On the other hand *P. pastoris* synthesizes large amounts of a homologous heme-containing catalase protein under alchohol oxidase (AOX) promoted recombinant expression. Thus gene expression under AOX promoter might be beneficial for MnP production due to the added need for heme synthesis inside the cells. *Coprinus cinereus* produces a class II excreted fungal peroxidase (CiP) that readily oxidizes phenols, but is unable to oxidize veratryl alcohol or Mn2+−ions (Hofrichter *et al.*^2010^). This enzyme is successfully produced in a recombinant process using *P. pastoris* (Kim *et al.*^2009^a) with high productivities (peroxidase activity over 1200 U ml-1 and total protein over 1.6 g l-1) and is also sold as a commercial enzyme by Novozymes (Baylase®). The productivity of this heme-containing peroxidase was optimized by host and expression promoter selection (Kim *et al.*^2009^b). Highest productivities were obtained by using the AOX promoter with a fast methanol utilization strain (Mut+) of *P. pastoris*. This supports the theory, that inducing the methanol utilization pathway in a production host can promote the recombinant production of heme-containing peroxidases. Commercially available MnP (from *Phlebia sp. Nf* b19) and VP (from *Bjerkandera adusta*) from Jena Bioscience GmbH are native enzymes and overly expensive for any kind of industrial use.

In this study several well known MnP enzymes were compared. MnP and laccase and their regulation in *P. rivulosus* has been well characterized byHakala *et al.* (2005&^2006^). The Differential regulation of MnP isoforms in *P. rivulosus* was also noted in these articles. In this study only one MnP from *P. rivulosus* is characterized, but this may very well be a group of MnP isoenzymes with approximately the same isoelectric points and molecular sizes. In characterization studies by Hildén *et al.* (2008), the MnP2 enzyme from *Phlebia sp. Nf* b19 showed a 96% amino acid identity to the MnP2 enzyme of *P. radiata* in the primary structure. In this study the specific activities and sizes of the MnP enzymes produced by these related fungal strains were in the same range. Even in growth and MnP production these strains showed similarities (extremely slow growth and late onset of MnP production). Hildén *et al.* (2005) describe two MnP enzymes from *P. radiata* that are different in their primary structure, intron amount, length and crystal structure. The other isoenzyme being structurally related to LiP, but having an alanine residue instead of tryptophan while still having a conserved Mn2+−binding site. In this study three MnPs from *P. radiata* were separated, but they seemed to be highly similar in size and specific activity for Mn2+ oxidation.

Many technical applications for MnP have been reported with promising results. The utilization of the enzyme is still dependent on a cost-effective recombinant production process and possibly the discovery of more robust novel isoenzymes or modifications of the currently known ones. Furthermore, optimization of enzymatic treatment processes for various technical lignins (Vishtal &Kraslawski 2011), paper pulps (Maijala *et al.*^2008^;Xu *et al.*^2010^) and organopollutants (Sack *et al.*^1997^) with proper process conditions and co-oxidants will probably increase the interest in MnP. Delignification of pinewood sawdust using a MnP treatment in this study was relatively inefficient. The use of several co-oxidants and other enzymes involved in biological ligninolysis may help to achieve more thorough enzymatic delignification demonstrated by many MnP producing and LiP-negative white-rot fungi (Hammel &Cullen 2008). In previous laboratory experiments byHofrichter *et al.* (1998) andKapich *et al.* (1999) Mineralization and solubilization of synthetic (14C-labeled) large molecular weight lignin by MnP has been reported. These and various other studies suggest that isolated MnP enzymes can be used to delignify biomasses. However, for now the utilization of class II peroxidases to degrade polluting substances in soils is technically more appealing.

## Competing interests

The authors declare that they have no competing interests.

## References

[B1] Bharagava RN Chandra R Rai V Isolation and characterization of aerobic bacteria capable of the degradation of synthetic and natural melanoidins from distillery effluent World J Microbiol Biotechnol 2009 25 737 744

[B2] Bonnarme P Jeffries T Mn(II) regulation of lignin peroxidases and manganese-dependent peroxidases from lignin-degrading white rot fungi Appl Environ Microbiol 1990 56 210 217 16348093 10.1128/aem.56.1.210-217.1990 PMC183274

[B3] Conesa A van den Hondel C Punt P Studies on the production of fungal peroxidases in Aspergillus niger Appl Environ Microbiol 2000 66 3016 3023 10877800 10.1128/aem.66.7.3016-3023.2000 PMC92105

[B4] Floudas D The Paleozoic origin of enzymatic lignin decomposition reconstructed from 31 fungal genomes Science 2012 336 6089 17151719 10.1126/science.1221748 22745431

[B5] Hakala T Lundell T Galkin S Maijala P Kalkkinen N Hatakka A Manganese peroxidases, laccases and oxalic acid from the selective white-rot fungus Physisporinus rivulosus grown on spruce wood chips Enzyme Microb Tech 2005 36 461 468

[B6] Hakala T Hildén K Maijala P Olsson C Hatakka A Differential regulation of manganese peroxidases and characterization of two variable MnP encoding genes in the white-rot fungus Physisporinus rivulosus Appl Microbiol Biotechnol 2006 73 839 849 17031639 10.1007/s00253-006-0541-0

[B7] Hammel K Cullen D Role of fungal peroxidases in biological ligninolysis Curr Opin Plant Biol 2008 11 349 355 18359268 10.1016/j.pbi.2008.02.003

[B8] Hatakka A Uusi-Rauva A Degradation of 14C-labelled poplar wood lignin by selected white-rot fungi Eur J Appl Microbiol Biotechnol 1983 17 235 242

[B9] Hildén K Martinez A Hatakka A Lundell T The two manganese peroxidases Pr-MnP2 and Pr-MnP3 of Phlebia radiata, a lignin-degrading basidiomycete, are phylogenetically and structurally divergent Fungal Genet Biol 2005 42 403 419 15809005 10.1016/j.fgb.2005.01.008

[B10] Hildén K Bortfeldt R Hofrichter M Hatakka A Lundell T Molecular characterization of the basidiomycete isolate Nematoloma frowardii b19 and its manganese peroxidase places the fungus on the corticioid genus Phlebia Microbiology 2008 154 2371 2379 18667569 10.1099/mic.0.2008/018747-0

[B11] Hofrichter M Vares T Scheibner K Galkin S Sipilä J Hatakka A Mineralization and solubilization of synthetic lignin by manganese peroxidases from Nematoloma frowardii and Phlebia radiata J Biotechnol 1999 67 217 228

[B12] Hofrichter M Lundell T Hatakka A Conversion of milled pine wood by manganese peroxidase from Phlebia radiata Appl Environ Microbiol 2001 67 4588 4593 11571160 10.1128/AEM.67.10.4588-4593.2001 PMC93207

[B13] Hofrichter M Scheibner K Schneegass I Fritsche W Enzymatic combustion of aromatic and aliphatic compounds by manganese peroxidase from Nematoloma frowardii Appl Environ Microbiol 1998 64 399 404 16349496 10.1128/aem.64.2.399-404.1998 PMC106057

[B14] Hofrichter M Review: lignin conversion by manganese peroxidase (MnP) Enzyme Microb Technol 2002 30 454 466

[B15] Hofrichter M Ullrich R Pecyna M Liers C Lundell T New and classic families of secreted fungal heme peroxidases Appl Microbiol Biotechnol 2010 87 871 897 20495915 10.1007/s00253-010-2633-0

[B16] Irie T Honda Y Watanabe T Kuwahara M Homologous expression of recombinant manganese peroxidase genes in ligninolytic fungus Pleurotus ostreatus Appl Microbiol Biotechnol 2001 55 566 570 11414322 10.1007/s002530000540

[B17] Jiang F Kongsaeree P Schilke K Lajoie C Kelly C Effects of pH and temperature on recombinant manganese peroxidase production and stability Appl Biochem Biotechnol 2008 146 15 27 18421583 10.1007/s12010-007-8039-5

[B18] Jiang F Kongsaeree P Charron R Lajoie C Xu H Scott G Kelly C Production and separation of manganese peroxidase from heme amended yeast cultures Biotechnol Bioeng 2008 99 540 549 17680655 10.1002/bit.21590

[B19] Jimenez-Tobon G Kurzatkowski W Rozbicka B Solecka J Pocsi I Penninckx M In situ localization of manganese peroxidase production in mycelial pellets of Phanerochaete chrysosporium Microbiology-Sgm 2003 149 3121 3127 10.1099/mic.0.26451-0 14600224

[B20] Johnson T Pease EA Li J Tien M Production and characterization of recombinant lignin peroxidase isozyme H2 from Phanerochaete chrysosporium using recombinant baculovirus Arc Biochem Biophys 1992 296 2 660 666 10.1016/0003-9861(92)90624-6 1632652

[B21] Kamitsuji H Honda Y Watanabe T Kuwahara M Production and induction of manganese peroxidase isozymes in a white-rot fungus Pleurotus ostreatus Appl Microbiol Biotechnol 2004 65 287 294 14767623 10.1007/s00253-003-1543-9

[B22] Kapich A Hofrichter M Vares T Hatakka A Coupling of manganese peroxidase-mediated lipid peroxidation with destruction of nonphenolic lignin Biochem Bioph Res Co 1999 259 1 212 219 Model Compounds and 14C-Labeled Lignins 10.1006/bbrc.1999.0742 10334942

[B23] Kim S Lee J Won K Kim Y Song B Functional expression of Coprinus cinereus peroxidase in Pichia pastoris Process Biochem 2009 44 7 731 735

[B24] Kim S Lee J Kim Y Song B Optimization of the functional expression of Coprinus cinereus peroxidase in pichia pastoris by varying the host and promoter J Microbiol Biotechn 2009 19 9 966 971 10.4014/jmb.0901.018 19809254

[B25] Lankinen P Hilden K Aro N Salkinoja-Salonen M Hatakka A Manganese peroxidase of Agaricus bisporus: grain bran-promoted production and gene characterization Appl Microbiol Biotechnol 2005 66 401 407 15538559 10.1007/s00253-004-1731-2

[B26] Li J Gellerstedt G Toven K Steam explosion lignins; their extraction, structure and potential as feedstock for biodiesel and chemicals Biores Technol 2009 100 2556 2561 10.1016/j.biortech.2008.12.004 19157871

[B27] Li D Youngs H Gold M Heterologous expression of a thermostable manganese peroxidase from Dichomitus squalens in Phanerochaete chrysosporium Arch Biochem Biophys 2001 385 348 356 11368016 10.1006/abbi.2000.2159

[B28] Maijala P Kleen M Westin C Poppius-Levlin K Herranen K Lehto JH Reponen P Mäentausta O Mettala A Hatakka A Biomechanical pulping of softwood with enzymes and white-rot fungus Physisporinus rivulosus Enzyme Microb Technol 2008 43 169 177

[B29] Martinez M RuizDuenas F Guillen F Martinez A Purification and catalytic properties of two manganese peroxidase isoenzymes from Pleurotus eryngii Eur J Biochem 1996 237 424 432 8647081 10.1111/j.1432-1033.1996.0424k.x

[B30] Mayfield M Kishi K Alic M Gold M Homologous expression of recombinant manganese peroxidase in Phanerochaete chrysosporium Appl Environ Microbiol 1994 60 4303 4309 7811070 10.1128/aem.60.12.4303-4309.1994 PMC201985

[B31] Mills M Payne S Genetics and regulation of heme iron transport in Shigella dysenteriae and detection of an analogous system in Echerichia coli O157:H J Bacteriol 1995 177 3004 3009 7768795 10.1128/jb.177.11.3004-3009.1995 PMC176986

[B32] Mishra M Thakur I Isolation and characterization of alkalotolerant bacteria and optimization of process parameters for decolorization and detoxification of pulp and paper mill effluent by Taguchi approach Biodegradation 2010 21 967 978 20401684 10.1007/s10532-010-9356-x

[B33] Moilanen A Lundell T Vares T Hatakka A Manganese and malonate are individual regulators for the production of lignin and manganese peroxidase isozymes and in the degradation of lignin by Phlebia radiata Appl Microbiol Biotechnol 1996 45 792 799

[B34] Moreira P Duez C Dehareng D Antunes A Almeida-Vara E Frère J Molecular characterisation of a versatile peroxidase from a Bjerkandera strain J Biotech 2005 118 4 339 352 10.1016/j.jbiotec.2005.05.014 16026883

[B35] Moreira M Feijoo G Canaval J Lema J Semipilot-scale bleaching of Kraft pulp with manganese peroxide Wood Sci Technol 2003 37 117 123

[B36] Moreira M Sierra-Alvarez R Lema JM Feijoo G Field J Oxidation of lignin in eucalyptus Kraft pulp by manganese peroxidase from Bjerkandera sp strain BOS55 Biores Technol 2001 78 71 79 10.1016/s0960-8524(00)00161-9 11265791

[B37] Nuske J Scheibner K Dornberger U Ullrich R Hofrichter M Large scale production of manganese-peroxidase using agaric white-rot fungi Enzyme Microb Technol 2002 30 556 561

[B38] Paice M Reid I Bourbonnais R Archibald F Jurasek L Manganese peroxidase, produced by Trametes-versicolor during pulp bleaching, demethylates and delignifies Kraft pulp Appl Environ Microbiol 1993 59 260 265 16348850 10.1128/aem.59.1.260-265.1993 PMC202088

[B39] Palma C Martínez A Lema J Martínez M Different fungal manganese-oxidizing peroxidases: a comparison between Bjerkandera sp. And Phanerochaete chry-sosporium J Biotechnol 2000 77 235 245 10682282 10.1016/s0168-1656(99)00218-7

[B40] Pease E Aust S Tien M Heterologous expression of active manganese peroxidase from Phanerochaete chrysosporium using the baculovirus expression system Biochem Biophys Research Comm 1991 179 897 903 1898410 10.1016/0006-291x(91)91903-p

[B41] Perie F Gold M Manganese regulation of manganese peroxidase expression and lignin degradation by the white-rot fungus Dichomitus-squalens Appl Environ Microbiol 1991 57 2240 2245 1768094 10.1128/aem.57.8.2240-2245.1991 PMC183557

[B42] Petruccioli M Frasconi M Quaratino D Covino S Favero G Mazzei F Federici F D’Annibale A Kinetic and redox properties of MnP II, a major manganese peroxidase isoenzyme from Panus tigrinus CBS 577.79 J Biol Inorg Chem 2009 14 1153 1163 19578878 10.1007/s00775-009-0559-8

[B43] Reading NS Aust SD Role of disulfide bonds in the stability of recombinant manganese peroxidase Biochemistry 2001 40 8161 8168 11434786 10.1021/bi010440i

[B44] Sack U Hofrichter M Fritsche W Degradation of polycyclic aromatic hydrocarbons by manganese peroxidase of Nematoloma frowardii FEMS Microbiol Lett 1997 152 227 234 9273311 10.1111/j.1574-6968.1997.tb10432.x

[B45] Schneegaβ I Hofrichter M Scheibner K Fritsche W Purification of the main manganese peroxidase isoenzyme MnP2 from the white-rot fungus Nematoloma frowardii b19 Appl Microbiol Biotechnol 1997 48 602 605

[B46] Singh D Zeng J Chen S Increasing manganese peroxidase productivity of Phanerochaete chrysosporium by optimizing carbon sources and supplementing small molecules Lett Appl Microbiol 2011 53 120 123 21535047 10.1111/j.1472-765X.2011.03070.x

[B47] Silva E Martins S Milagres A Extraction of manganese peroxidase produced by Lentinula edodes Biores Technol 2008 99 2471 2475 10.1016/j.biortech.2007.04.064 17583498

[B48] Sklenar J Niku-Paavola M-L Santos S Man P Kruus K Novotny C Isolation and characterization of novel pI 4.8 MnP isoenzyme from white-rot fungus Irpex lacteus Enzyme Microb Technol 2010 46 550 556

[B49] Steffen K Hofrichter M Hatakka A Purification and characterization of manganese peroxidases from the litter-decomposing basidiomycetes Agrocybe praecox and Stropharia coronilla Enzyme Microb Technol 2002 30 550 555

[B50] Stewart P Whitwam R Kersten P Cullen D Tien M Efficient expression of a Phanerochaete chrysosporium manganese peroxidase gene in Aspergillus oryzae Appl Environ Microbiol 1996 62 860 864 8975615 10.1128/aem.62.3.860-864.1996 PMC167852

[B51] Sundaramoorthy M Kishi K Gold M Poulos T Crystal structures of substrate binding site mutants of manganese peroxidase J Biol Chem 1997 272 17574 17580 9211904 10.1074/jbc.272.28.17574

[B52] Sutherland G Zapanta L Tien M Aust S Role of calcium in maintaining the heme environment of manganese peroxidase Biochemistry 1997 36 3654 3662 9132018 10.1021/bi962195m

[B53] Susla M Novotny C Erbanova P Svobodova K Implication of Dichomitus squalens manganese-dependent peroxidase in Dye decolorization and cooperation of the enzyme with laccase Folia Microbiol 2008 53 479 485 19381471 10.1007/s12223-008-0075-1

[B54] Swamy J Ramsay J Effects of Mn2+ and NH4+: concentrations on laccase and manganese peroxidase production and Amaranth decoloration by Trametes versicolor Appl Microbiol Biotechnol 1999 51 391 396

[B55] Taboada-Puig R Lú-Chau T Moreira M Feijoo G Martínez M Lema J A new strain of Bjerkandera sp. production, purification and characterization of versatile peroxidase World J Microbiol Biotechnol 2011 27 115 122

[B56] Tien M Kirk T Lignin peroxidase of Phanerochaete chrysosporium Methods Enzym 1988 161 238 248

[B57] Urek R Pazarlioglu N Purification and partial characterization of manganese peroxidase from immobilized Phanerochaete chrysosporium Process Biochem 2004 39 2061 2068

[B58] Vares T Kalsi M Hatakka A Lignin peroxidases, manganese peroxidases, and other ligninolytic enzymes produced by Phlebia radiata during solid-state fermentation of wheat straw Appl Environ Microbiol 1995 61 3515 3520 16535139 10.1128/aem.61.10.3515-3520.1995 PMC1388701

[B59] Vishtal A Kraslawski A Challenges in industrial applications of technical lignins BioResources 2011 6 3547 3568

[B60] Wang P Hu X Cook S Begonia M Lee K Hwang H-M Effect of culture conditions on the production of ligninolytic enzymes by white rot fungi Phanerochaete chrysosporium (ATCC 20696) and separation of its lignin peroxidase World J Microbiol Biotechnol 2008 24 2205 2212

[B61] Wang Y Vazquez-Duhalt R Pickard M Effect of growth conditions on the production of manganese peroxidase by three strains of Bjerkandera adusta Can J Microbiol 2001 47 277 282 11358165 10.1139/w01-007

[B62] Wariishi H Valli K Gold M Manganese(II) oxidation by manganese peroxidase from the basidiomycete Phanerochaete chrysosporium - kinetic mechanism and role of chelators J Biol Chem 1992 267 23688 23695 1429709

[B63] Whetten R Sederoff R Lignin biosynthesis Plant Cell 1995 7 1001 1013 12242395 10.1105/tpc.7.7.1001 PMC160901

[B64] Whitwam R Tien M Heterologous expression and reconstitution of fungal Mn peroxidase Arch Biochem Biophys 1996 333 439 446 8809085 10.1006/abbi.1996.0413

[B65] Whitwam R Gazarian I Tien M Expression of fungal Mn peroxidase in Escherichia coli and refolding to yield active enzyme Biochem Biophys Res Commun 1995 216 1013 1017 7488173 10.1006/bbrc.1995.2721

[B66] Xu H Scott G Jiang F Kelly C Recombinant manganese peroxidase (rMnP) from Pichia pastoris. Part 1: Kraft pulp delignification Holzforschung 2010 64 137 143

[B67] Xu H Scott G Jiang F Kelly C Recombinant manganese peroxidase (rMnP) from Pichia pastoris. Part 2: application in TCF and ECF bleaching Holzforschung 2010 64 145 151

[B68] Yadav S Chandra R Rai V Characterization of potential MnP producing bacteria and its metabolic products during decolourisation of synthetic melanoidins due to biostimulatory effect of D-xylose at stationary phase Process Biochem 2011 46 9 1774 1784

